# Scaling and Predictability in Stock Markets: A Comparative Study

**DOI:** 10.1371/journal.pone.0091707

**Published:** 2014-03-14

**Authors:** Huishu Zhang, Jianrong Wei, Jiping Huang

**Affiliations:** Department of Physics and State Key Laboratory of Surface Physics, Fudan University, Shanghai, China; University of Warwick, United Kingdom

## Abstract

Most people who invest in stock markets want to be rich, thus, many technical methods have been created to beat the market. If one knows the predictability of the price series in different markets, it would be easier for him/her to make the technical analysis, at least to some extent. Here we use one of the most basic sold-and-bought trading strategies to establish the profit landscape, and then calculate the parameters to characterize the strength of predictability. According to the analysis of scaling of the profit landscape, we find that the Chinese individual stocks are harder to predict than US ones, and the individual stocks are harder to predict than indexes in both Chinese stock market and US stock market. Since the Chinese (US) stock market is a representative of emerging (developed) markets, our comparative study on the markets of these two countries is of potential value not only for conducting technical analysis, but also for understanding physical mechanisms of different kinds of markets in terms of scaling.

## Introduction

Stock markets provide an opportunity for people to increase their wealth. Most of people who invest in stock markets want to earn excess profits [1]. So various technical trading strategies based on moving averages, Bollinger lines and so on have been set up [2]–[4]. However it is shown that even the experienced fund managers can’t ensure the abnormal returns [5], let alone the individual investors [6]. Biondo (2013) compared four mainstream technical strategies including the random one, and obtained the following results: the average percentages of wins for these strategies are similar, but the risk of the random one is surprisingly the lowest [7]. We all know that the stock markets belong to the family of complex systems. Not only many real systems in physics [8], [9] but also lots of others like geophysical, social and biological systems are part of them. Agent-based modeling is an important way to analyze the behavior of agents in complex systems [10], [11], which has been also widely used in analyzing stock markets [12], [13].

The strength of predictability of time series is a significant property for one to design a trading strategy, so many methods like Box-Jenkins methodology [14] and Bayesian model averaging [15] have been created to analyze the predictability of time series. So far several relevant works have been done. For example, Fama and French (1989) found that conditional expected returns vary with the business cycles because rational agents smooth consumption over time [16]. Burton (2004) reviewed the success of past studies to predict future equity returns, and found that although some predictability of returns exists, there is no evidence of any systematic inefficiency that would ensure investors to earn excess returns [17]. Venkat (2004) found that annual excess returns on the stock market index are negatively related to the returns of glamour stocks during the 2001–2004 period, which means there existed some predictability [18]. In 2012, Grönlund, Yi and Kim found that the local maxima in the profit landscape are spread in the form of a fractal structure, that it shows the stock market has low predictability by technical analysis [19].

It is well known that the US stock market has a long history, which can be traced back to the period of the War of Independence. Nowadays, the US stock market has grown up as the most developed and mature stock market in the world. Comparing with the US stock market, the Chinese stock market is much more emerging and just precedes a quarter of a century. [20]. According to the Efficient Markets Hypothesis, the more developed market which is more efficient has weaker predictability by technical analysis [21]. Is the emerging market of China really more predictable than the developed market of US? In this work, we will use the profit landscape to compare the predictability of indexes and individual stocks in Chinese and US stock markets.

## Methods

To proceed, we resort to the profit landscape method [19] for the following data processing. Meanwhile, one of the most basic trading strategies will be utilized to make the profit landscape. The trading decision at time 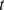 only depends on the log return 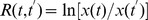, in which 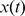 and 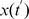 is the daily closing price at time 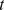 and 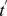 with 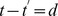 (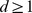). Next is this simple trading strategy.

- If *R*(*t*,*t*′)≥*p*/*K*, *f_s_* fraction of stocks in account are sold. And *K* is the scale factor.
- If *R*(*t*,*t*′)≤−*q*/*K*, one spends *f_c_* fraction of cash buying the stocks at current price.

Here 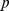 and 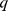 are two parameters that determine in which condition one should buy or sell. For convenience, we just define that 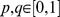. Then the detailed parameters for performing our simulations are as follows: (i) At the first trading day, one gain initial cash 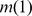 and initial number 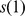 of stocks. We set initial money 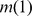 as 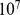 and initial stocks *s*(1) as 0. Moreover, 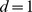 (the value of 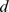 hardly influence the results [19]); (ii) In real stock markets, the minimum trading volume exists. We set this minimum volume as 1 for convenience. One can imagine that no difference will appear if we increase this volume and proportional increase the initial cash as well. (iii) We also can’t ignore the transaction tax in real market, thus we assume that this tax rate is 0.1%, the same as the Chinese securities transaction tax rate; (iv) The two proportion parameters, 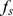 and 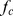, rarely affect the exponent which we care about in our study. Thus in order to facilitate the model, they can just be set as 0.5. (v) 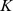 is the scale factor that will be adjusted in different data series and explained concretely soon after.

For given values of 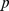, 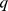, the strategy is repeatedly executed till the last day 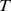. For the sake of simplicity, we assume that the risk-free interest rate is zero, and the cash dividends provided by companies are neglected. Then, we can assess the performance of strategy only by final profit defined by 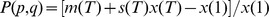. In order to plot the profit landscape, we’d like to traverse almost all possible 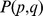. Therefore, We define parameter 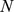 as the resolution to determine the minimum variation amount 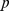 and 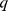. Then the 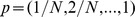 and 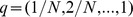. According to the various combinations of 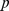 and 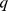, we can gain 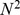 values of 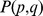. Then we can plot a three-dimensional figure called profit landscape through 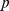, 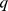, and 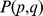.

When the resolution 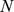 changes, the appearance and scale of profit landscape also changes. So we can obtain the relationship between 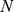 and the number of local maxima, 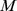, on landscape. The local maxima are these points whose values are larger than the four neighboring ones. If one wants to optimize the investment strategies, he or she will establish strategies within a certain range of peaks on profit landscape. Hence, nobody hopes that the locations and amounts of peaks change a lot when 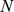 increases. It is quite clear that if a price series is completely periodic or regular, the best strategies will be determinate. The peaks will be certain points or small platforms on profit landscape and their locations will never change when 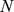 increases. In this case, one will have algebraic form of 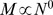 (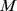 does not vary with 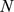), so the technical strategies are highly easy to be formulated. If 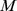 increases with 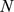 following 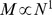, it means that the new local maxima only appear along horizontal or vertical axis on the landscape when 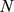 increases, so it will not be too difficult for one to optimize strategies (either 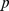 or 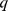 is more likely to be optimized). And in case we choose the unpredictable random series (say, geometric Brownian motion [22]) to do the above analysis, we will obtain algebraic form of 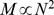 [19]. In this situation, no parameters can be optimized and the technical strategies are invalid. To sum up, one can easily see that the predictability of price series is stronger when the exponent of power-law is smaller. In this article, we gain the relation between 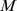 and 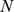 following the power-law distribution with exponents all ranging from 1 to 2. These results are consistent with the fact that real price movements cannot be totally described by random series [23], [24].

**Figure 1:**
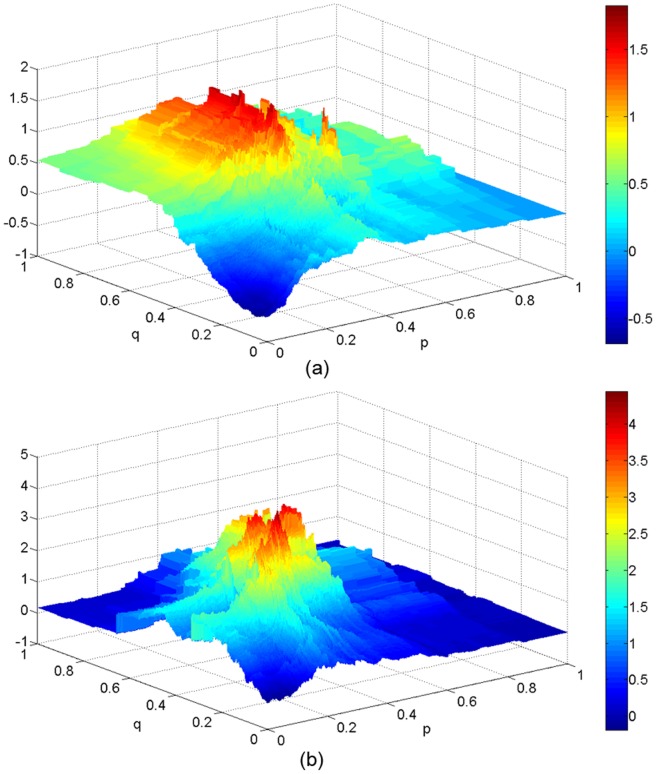
Two model profit landscapes in the two dimensional space for (a) the Shanghai Composite Index and (b) one component stock in the S&P500 Index. Parameters: *K* = 10 and *N* = 1000.

Then we’d like to explain the meaning about scale factor 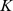. It can be easily seen that the amount of computation for simulations is directly proportional to 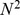. So when 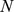 is large, the amount of the computation is extremely tremendous. Parameter 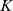 is the scale factor for zooming in the profit landscape as if we use a magnifier to observe it. Given example, if 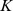 = 10, then the 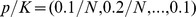. As a result, this parameter can help to increase precision even 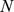 is not large enough. In addition, if 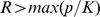, some strategies will fall out the landscape resulting the imperfectly effective optimization. Consequently, 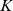 should be adjusted to confirm that 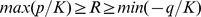, at the same time the landscape contains all the possible strategies. Fig. 1 shows two figures of the profit landscape with certain value of 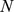 and 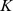.

## Results

In this work, we use daily closing price data of Standard & Poor’s 500 Index (S&P500 Index; a representative stock index in US stock markets) and the Shanghai Composite Index (a representative stock index in Chinese stock markets)from 1997 to 2012. We also choose daily closing price data of the 80 earliest listed stocks from Standard & Poor’s 500 during 1997 to 2012. The chief reasons why we select these companies as symbol of US stocks involve two factors. One attribute to their long histories, and the other is that these companies belong to various industries. At the same time, the daily closing price data of all the 34 stocks (listed before 1997) from the Shanghai Stock Exchange during the same period are chosen for analysis.

**Figure 2:**
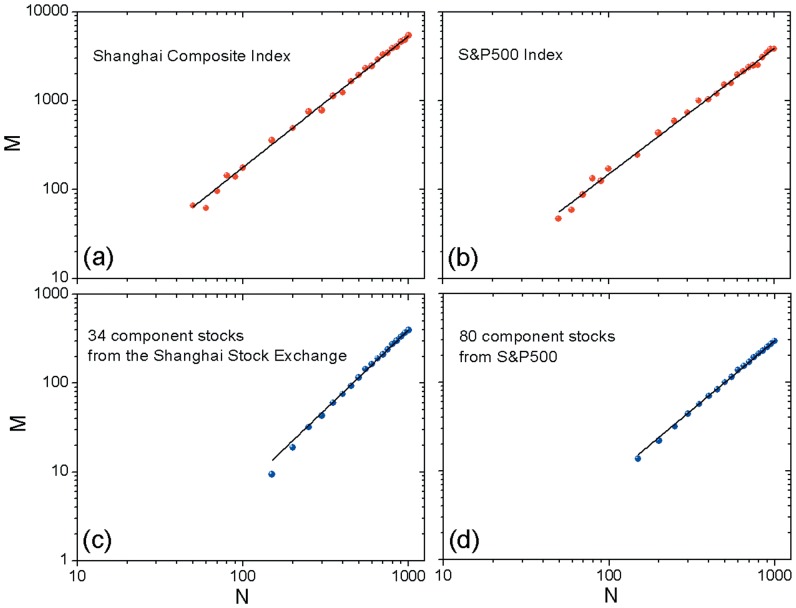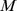 versus 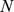 for (a) the Shanghai Composite Index (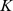 = 10), (b) the S&P500 Index (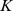 = 10), (c) 34 component stocks from Shanghai Stock Exchange (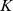 = 1), and (d) 30 component stocks from S&P500 (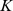 = 1). The relation between 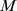 and 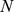 follows the power-law distribution(as indicated by the straight line in each panel) with exponent (a) 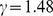 for 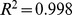, (b) 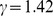 for 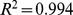, (c) 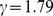 for 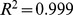, and (d) 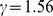 for 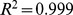. Here 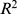 denotes the regression coefficient that represents the degree of fitting with the power law: the perfect fitting corresponds to 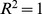 [26].

We first analyze the indexes of both China and US. There is no doubt that the Shanghai composite index floats within plus or minus 10% in one day. Simultaneously, though the S&P500 Index does not have such limitation, the maximum daily price fluctuation from 1997 to 2012 is only a little more than 10%. So in order to enhance the accuracy of the result, we can set 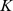 as 10. In this situation, the maximum 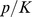 and 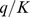 is 0.1, which is approaching to the maximum value of 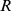 (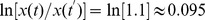). Therefore, the profit landscape can has completeness and high precision as well. Fig. 2(a,b) shows the relation between 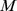 and 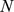 of the landscape from two indexes. Clearly, we obtain the power-law distribution of 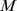 versus 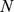 with the exponent 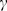 equaling 1.48 for the Shanghai Composite Index and 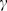 equaling 1.42 for S&P500 Index, respectively. In view of the presence of fitting error, we can conclude that the predictability of the Shanghai Composite Index is approximately equal to that of S&P500 index.

Then we analyze the individual stocks which are components of these two indexes. Owing to the daily price fluctuation of several stocks in S&P500 are enormous, we just set 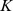 as 1 to ensure that the landscape contains almost all the possible conditions. First of all, we use the above-mentioned method to get every 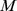 of component stocks. After that, we calculate the average of 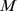 and utilize it to analyze the result for individual stocks; see Fig. 2(c,d) what shows relation between 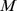 and 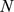 of the landscape from individual stocks. Also, we observe power-law distribution of 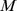 versus 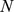 with the exponent 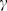 equaling 1.79 for the stocks of Chinese companies and 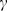 equaling 1.56 for US ones. That is, the predictability of individual stocks of the Shanghai Stock Exchange is lower than that of S&P500. On the other hand, comparing Fig. 2(a,b) with Fig. 2(c,d), we can also see that the value of exponent of index [in Fig. 2(a,b)] is smaller than that of individual stocks [in Fig. 2(c,d)] in both two markets. Therefore, it appears to be more difficult for one to predict the individual stocks than indexes in both Chinese and US stock markets.

## Discussion

We have studied the profit landscape defined by some straight-forward investment strategies in this work. The results show that the relation between resolution 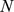 and the number of local maxima 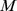 on the profit landscape follows the power-law distribution, and the value of power-law exponent can be used to characterize the predictability of the price series. From the results, we can conclude that the predictability of Shanghai Composite Index is roughly the same as that of S&P500 Index. However, the predictability of individual stocks does not give the similar results: the predictability of individual stocks in Shanghai Stock Exchange is lower than that in S&P500. It is well known that the Chinese government sometime has a great influence on stock markets, and the black-box operation exists in the exchanges of some individual stocks. So we suppose these two reasons play a crucial role in this result.

On the other hand, we can also conclude that the predictability of stock indexes is higher than individual stocks in both the Shanghai Composite Index and S&P500 Index. We know that the index is calculated by the individual stocks. In a price-weighted index such as the Dow Jones Industrial Average, the price of each component stock is the only consideration when determining of the index. S&P500 Index or the Shanghai Composite Index is a capitalization-weighted index whose components are weighted according to the total market capitalizations of their circulating shares. So, if the price of one stock has some fluctuations for unknown reason, these changes will hardly influence the index. On the contrary, if the price of index varies greatly someday, lots of the individual stocks follow the similar trend with the index. Therefore, the weighted averaging operation of index enhances its predictability. We know that the price series in long time scales are approximate as a random process [25], so the predictability is not high as expected. We plan to study the profit landscape of time series in short time scales like high frequency price series and so on.

Finally, the comparative study reported in this work is of potential value not only for conducting technical analysis, but also for understanding physical mechanisms of different kinds of markets in terms of scaling, being beyond the efficient markets hypothesis.
